# Development of an Optical Sensor for Real-Time Monitoring of Hemodynamic Parameters in Extracorporeal Settings

**DOI:** 10.1109/JTEHM.2026.3653633

**Published:** 2026-01-12

**Authors:** Osama Elgabori, William B. Scammon, Kelly R. Strong, Jingyi Wu, Keith E. Cook, Jana M. Kainerstorfer

**Affiliations:** Department of Biomedical Engineering Pittsburgh PA 15213 USA; Neuroscience Institute Pittsburgh PA 15213 USA

**Keywords:** Extracorporeal systems, hemodynamic monitoring, medical devices, optical spectroscopy

## Abstract

Objective: Whole blood oxygen saturation and hemoglobin concentration are key markers of health across a variety of clinical contexts. Extracorporeal systems (dialysis, cardiopulmonary bypass, ECMO, etc.) require close monitoring of these parameters for proper patient treatment and intervention. Currently, blood gas analyzers are the gold standard for such measurements, however, these devices are invasive and fail to provide real-time results. In contrast, optical sensors can non-invasively probe whole blood for real-time monitoring of oxygen saturation and hemoglobin concentration. While commercial devices that implement such sensors exist, they not only fail to reduce the overall footprint of extracorporeal systems but instead increase it. Technology or Method: In this work, we develop small form factor optical sensors to be compatible with extracorporeal systems and obtain accurate real-time results using an empirical calibration method. We evaluate the performance of a pair of these optical sensors using this calibration through in-vitro experiments with whole blood. Results: Results showed an average accuracy root-mean square error of 1.30 g/dL for hemoglobin concentration and 4.76 % for saturation. Conclusions: These results demonstrate the potential viability of these sensors for use in assessing extracorporeal device performance and patient health.

Clinical and Translational Impact: The proposed sensors are lightweight and can be directly integrated into ECMO systems for monitoring hemoglobin and oxygen levels. Results are promising with device accuracy comparable to current commercial devices.

## I. Introduction

As a vital component in metabolism, oxygen must be reliably transported to tissue. Blood serves as the medium for oxygen transport, wherein oxygen is bound to hemoglobin within red blood cells. As such, accurate measurements of whole blood oxygen saturation (SO2) and total hemoglobin (Hgb) concentration are critical in a variety of clinical situations (e.g. anemia) [1], [2]. This need for accurate measurements is especially important in the context of extracorporeal systems like dialysis, cardiopulmonary bypass, and extracorporeal membrane oxygenation (ECMO) that require regular monitoring of vital physiological parameters to ensure proper patient treatment and management [3].

Benchtop blood gas analyzers are the gold standard for measurement of patient oxygen saturation and hemoglobin levels [4], [5], [6]. However, this method is time-consuming, requires invasive blood sampling and does not allow for continuous monitoring [7]. These drawbacks limit physicians’ ability to implement therapeutic changes in a timely fashion. Thus, there is a need to develop non-invasive instrumentation that allow for real-time accurate measurements. One method that is widely utilized for such measurements is optical spectroscopy [8], [9].

Optical spectroscopy is a non-invasive technique that examines the interaction of light with tissue for extraction of biochemical information. Depending on the wavelength of light used and signal of interest, there are a variety of optical methods that can be used for diagnostics [10]. For instance, absorption spectroscopy measures the attenuation (optical density) of light through tissue for determination of chromophore (absorber) concentrations [10], [11], [12].

For whole blood, hemoglobin is the primary absorber of light. The optical properties of hemoglobin change with oxygenation. Using at least two wavelengths, one can differentiate oxygenated and deoxygenated hemoglobin. For homogenous solutions, the absorption coefficient and thus concentration can be calculated by means of the Beer-Lambert Law (BLL). The equation assumes homogeneity as well as the solution being purely absorbing (no scattering). In the case of blood, however, solutions cannot be assumed to be purely absorbing. As hemoglobin is encapsulated within erythrocytes (red blood cells), a mismatch in refractive index occurs between the membrane and blood plasma resulting in light scattering [13], [14], [15]. This scattering causes additional attenuation of light separate from absorption that prevents the use of the BLL, making extraction of whole blood optical properties (and by extension saturation and Hgb concentration) challenging. Often light scattering in tissue can be addressed through diffuse optical methods, however, these approaches require that the scattering is an order of magnitude (at minimum) greater than absorption [16]. In the case of whole blood, scattering is approximately on the same order of magnitude as absorption known as the sub-diffuse (multiple scattering) regime, and thus description of light propagation in a diffuse medium is not applicable [17].

Various approaches to overcome the influence of sub-diffuse blood scattering for extraction of hemoglobin concentration and oxygen saturation have been proposed. These include Twersky’s multiple scattering theory for biological suspensions (e.g. blood) which provides a mathematical formulation that gives the total attenuation of light incident through a suspension of large, low-refracting, and absorbing particles [13], [15], [18], [19]. Modified photon diffusion models have also been developed to delineate absorption and scattering in whole blood for optical property extraction [13], [20], [21]. These approaches are complex, making them difficult to implement algorithmically. Additionally, they often require accurate knowledge of input light intensity, detector size and aperture, and prior knowledge of the geometry. In contrast, empirical calibration approaches that are simple to implement have been utilized allowing for direct extraction of hemoglobin saturation and concentration [22], [23], [24], [25], [26].

Our goal here is to develop optical sensors that provide real-time measurements of oxygen saturation and total hemoglobin concentration in whole blood using an empirically developed calibration. The goal of developing such sensors is to assess the functionality of ECMO gas exchangers and patient health with a portable and light-weight device unlike clinically available monitors. By clamping a pair of these sensors to the blood-filled gas exchanger inlet and outlet tubing, oxygen transfer can be assessed in real-time. We aim to assess the accuracy of these sensors using the empirical calibration method developed and assess their potential for clinical use in extracorporeal system settings for direct extraction of hemoglobin saturation and concentration.

The proposed system is advantageous over current clinical monitors (e.g. Spectrum M4 monitor and Terumo CDI 500 [4], [5]) as it is designed to integrate with both standard ECMO systems and newly developed wearable ECMO systems. Typically, ECMO is bulky and the associated monitoring equipment is not optimized in terms of footprint. Wearable devices like pulmonary assistant systems (PAS) [27]—consisting of an axial flow pump and a small biocompatible gas exchanger—provide the benefit of supporting patients (e.g. when performing ambulation) with reduced staff while still maintaining the same standard of care. The portability aspect of wearable ECMO necessitates that the associated bulky monitoring equipment, such as the hemoglobin and oxygen saturation monitors, be reduced in size as well. The Spectrum M4 monitor weighs 4.5 kg and has a 26.4 cm display and the Terumo CDI 500 weighs 2.8 kg with a $28\times 32 \times 15$cm display. Our work here improves upon these monitors through the development of a miniaturized optical monitor system. By addressing this need, our sensors can allow wearable ECMO systems to be translated clinically.

## II. Methods

### A. Hardware

**FIGURE 1.**
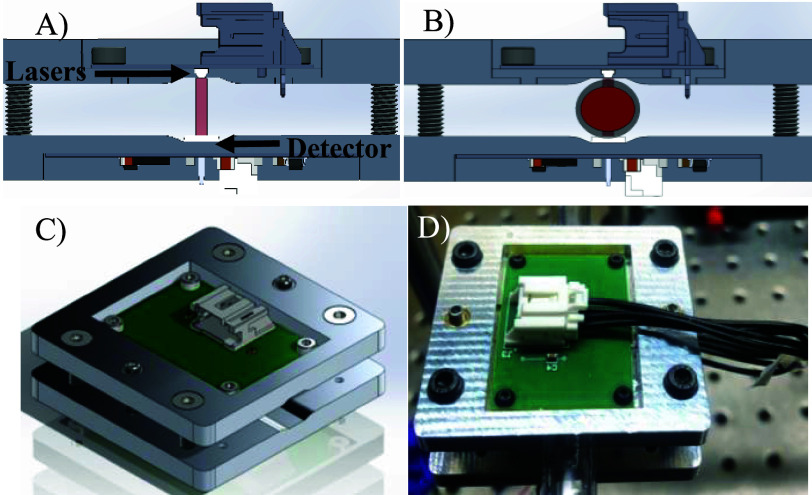
Axial cross-sections of the optical sensor (A, B)and its enclosure in full view (C, D). The four corner screws are used to compress the tubing (2 mm compression) with the center screws acting as a lock to prevent any further compression. This configuration ensures stability and fixed placement of the device.

Our optical sensors are based on transmission of light through a blood-filled tube. For illumination, two vertical cavity surface emitting lasers with wavelengths at 680 nm (VD-0680C-005M-1A-2A0, BrightLaser, Hong Kong) and at 850 nm (VD-0850C-010M-1A-2A0, BrightLaser, Hong Kong) were used. A 7 mm^2^ photodiode (BPW34, ams OSRAM, Premstätten, Austria) was used to measure light transmitted through the tube. A photometric analog front end (ADPD1080, Analog Devices, Massachusetts, United States) was used to control laser pulse timing and readout of photodiode signals. The components were placed on a split printed circuit board (PCB) for transmission measurements and housed on an aluminum enclosure. The enclosure is designed to clamp on to standard medical-grade Tygon tubing avoiding the need for custom connector making device translation simpler. Additionally, it is lightweight, weighing less than 50 g and of small form factor, measuring $4.8\times 5$ cm. These parameters are a significant reduction in size compared to clinical monitors as the smaller of the two devices weighs 2.8 kg with its smallest dimension being 15 cm. This aspect is useful for translation of the developed devices into existing ECMO systems and enabling translation of wearable devices. A cross-sectional layout of the device is shown in Figure 1A and 1B and the full enclosure design and developed sensor are shown in Figure 1C and 1D respectively.

**FIGURE 2.**
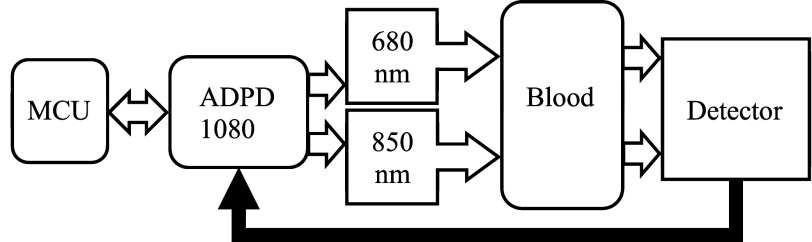
Block diagram of the sensor measurement. The arduino microcontroller (MCU) programs the ADPD1080, which stimulates the lasers and measures the return signal from the photodiode sequentially. The measurements are then sent to Arduino and reported to the user on a laptop.

The ADPD1080 front-end was programmed via a microcontroller (Arduino Nano ESP32, Arduino, UK) in the Arduino Integrated Development Environment using an open-source custom library developed for this specific integrated circuit [28]. This front-end was used to stimulate the lasers using $3~\mu $s pulses and measure the return signal from the photodiode through the output of a transimpedance (TIA) with a gain of 50 k $\Omega $ (Figure 2). The laser pulses are summed (maximum 127 pulse number) using an integrator to increase signal-to-noise ratio. The output voltage from the TIA is sent through a built-in bandpass filter (optimized for 2- $3~\mu $s pulses to remove the influence of ambient light) and is converted by the analog-to-digital converter (ADC) to an ADC value. The ADC intensity values scale linearly with the pulse number up to a maximum value of 65535. The currents for the 680 nm and 850 nm lasers were set to 9.97 mA and 18.41 mA respectively. These currents were selected to be above 80% of their maximum current to obtain maximal signal through the blood-filled tube (3/8” ID) but still prevent degradation of the lasers due to overdriving. To ensure consistent placement and stability of the sensors, adjustment screws (with a standard pitch tolerance of 20 um) were incorporated in the aluminum enclosure to apply a 2 mm compression to the tubing. As excess compression of the tubing will result in improper blood flow which could harm the patient, we examined different levels of compression to determine a safe level for a clinical case. This type of assessment can be performed by examining changes in blood flow resistance (calculated as a pressure difference divided by the volumetric flow rate). We found that compression up to a maximum of 4 mm will result in less than 1 mmHg $\cdot $min $\cdot $L^−1^ increase in blood flow resistance (data not shown). Typical ECMO circuits will have blood resistances that are an order of magnitude above these changes. We opted to be conservative in our compression of the tubing and thus selected half of the maximum level of compression (2 mm). According to the manufacturer specifications, contact with the sealant on the surface of the lasers can result in a distorted optical angle of the laser spot (i.e. not perpendicular to the surface) and damage the diode. As such, nylon spacers were added to the laser PCB to prevent contact between the surface of the lasers and the tubing. Other factors such as tubing curvature and the air gap between the lasers and tubing can result in light deflection as well. In cases where theoretical models are utilized, accounting for geometric factors is crucial [13], [20], [21], [29], [30]. However, we consider the impact of these factors to be minor due to our calibration-based approach. We assume these geometric factors are fixed by the enclosure and accounted for by the calibration. We computed the mean absolute difference (MAD) of the blood gas measurements and the readings from our sensors across seven days from validation data (eq. 1),

\begin{equation*} \mathrm {MAD =}\frac {\sum \nolimits _{i=1}^{n} \left |{{ s_{i}-b_{i} }}\right | }{n} \tag {1}\end{equation*}

where s_i_ is the predicted measurement from a given sensor and b_i_ is the corresponding blood gas measurement. Small variations in the day-to-day differences that are not statistically significant will enable us to conclude that geometric variations, such as tubing curvature, can be treated as negligible. For our statistical approach, we opted to fit a linear mixed effects model using MATLAB’s fitlme function with ‘day’ as the factor to handle cases of unequal observations between days. Furthermore, we developed two of these sensors to assess the reproducibility of our design and the results.

### B. Data Collection

**FIGURE 3.**
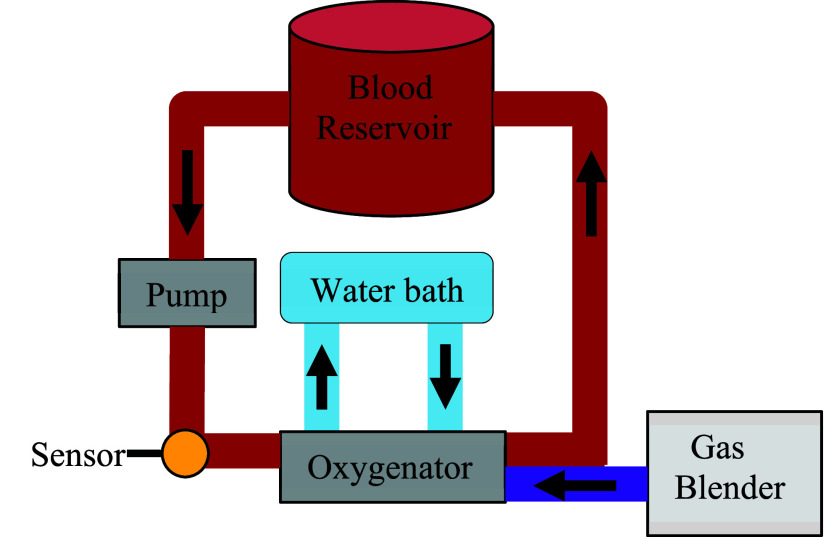
Schematic of the in-vitro recirculating blood circuit.

These sensors were calibrated and validated using a recirculating flow circuit (Figure 3) with 600 mL of bovine blood (7200801, Lampire Biological Labs, United States) at different hemoglobin concentrations and oxygen saturations. Prior to loading the blood in the circuit, the hemoglobin concentration was assessed via blood gas analysis and, if necessary, adjusted to the desired level. This adjustment was achieved by centrifuging the blood to remove plasma thereby increasing hematocrit and/or saline dilution to decrease hematocrit. The circuit contained a centrifugal blood pump (PediMag, Abbott Cardiovascular), an oxygenator (Terumo, Capiox FX 15 oxygenator), a sampling port, and a 400-ml soft bag reservoir for blood. The temperature of the blood was maintained at $37~\pm ~2^{\circ }$C using a water bath and the built-in heat exchanger of the oxygenator.

**FIGURE 4.**
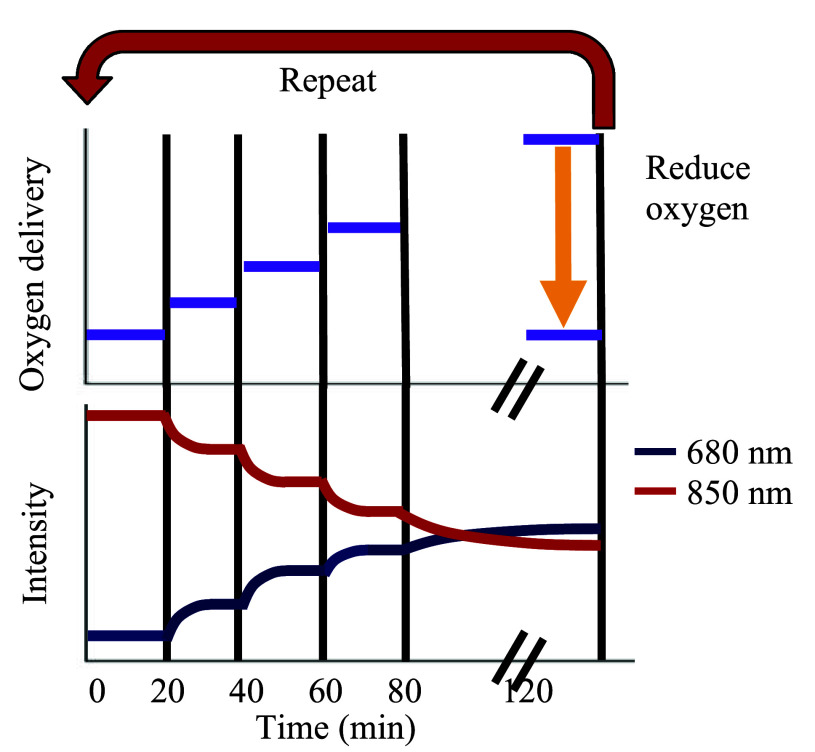
An illustration of the time course of experiment. The purple lines indicate the oxygen delivery (partial pressure) to the circuit, the blue line is the 680 nm laser intensity and red line is the 850 nm laser intensity. At 20-minute intervals, the oxygen saturation of the blood is increased. Prior to this change, the intensity data is collected from the sensors and a sample drawn from the circuit for ground-truth measurements. At 100% blood oxygen saturation the oxygen is reduced, and saline is used to dilute blood for repeated measurements at different hemoglobin concentrations.

The gas mixture contained oxygen, carbon dioxide, and nitrogen, and the concentrations of the gases were adjusted to achieve different levels of oxygen saturation for a given hemoglobin concentration. As shown in figure 4, for each gas change (purple lines), blood is allowed to circulate for 20 minutes for a measurable change in saturation to occur and stabilize. This increase in oxygenation results in a corresponding increase in intensity at 680 nm (blue line) and a decrease in intensity at 850 nm (red line). The stabilization of the blood optical properties (i.e. saturation and concentration) is directly related to the optical response settling time of our system. To avoid any settling time of the blood, we opted to wait twenty minutes after changing blood parameters before taking data. There is an initial turn on settling time for the sensors. However, the optical signals typically stabilize within a few minutes, well within the settling time of the blood. Once the blood saturation is stable, a sample is then drawn from the circuit to obtain ground truth blood oxygen saturation and hemoglobin concentration using a blood gas analyzer (BGA, ABL 825 Flex, Radiometer, Denmark). The optical sensors were clipped on the circuit’s tubing (3/8” ID x 9/16” OD x 3/32” Wall, ND 100-65 Tygon) and light transmission data were collected for each of the oxygenation states. During each blood sampling point, we collected one minute of intensity data with a 20 Hz sampling rate. Once blood is fully saturated, high flow nitrogen is used to reduce the oxygen saturation, and the saline is added to circuit for dilution to a lower hemoglobin concentration. This step enables additional saturation measurements at multiple hemoglobin concentrations.

### C. Data Processing: Calibration Approach

Using the collected in-vitro intensity data, we developed an empirical calibration method for extraction of hemoglobin concentration and oxygen saturation. As discussed in section B, the ADPD front-end must be programmed with the pulse number (the number of times to pulse the lasers prior to summing the ADC output) at the start of the experiment. The raw intensity data from the two sensors are first normalized by this preset pulse number. This parameter is adjusted to prevent low signal (at high hemoglobin concentrations) and saturation (at low hemoglobin concentrations) at the detector. We then computed the average normalized intensity of the sensors at each wavelength and their ratio to be used as inputs to the empirical calibration. This approach was taken to account for manufacturing differences in the sensors enabling the use of a single calibration for both sensors with appropriate scaling factors applied. We determined hemoglobin concentration based on a sparse regression-based model, with input parameters being the logarithm of the intensities and their ratio (eq. 2),

\begin{align*} \mathrm {Hgb}& =a_{1}{(\ln {I^{680})}}^{2}+a_{2}{(\ln {I^{850})}}^{2}+a_{3}R^{2}+a_{4}\ln I^{680} \\ & \quad +a_{5}\ln I^{850}+a_{6}R+a_{7}\ln I^{680}\ln I^{850} \\ & \quad +a_{8}\ln I^{680}R+a_{9} \tag {2}\end{align*}

where $I^{680}$ and $I^{850}$ are the scaled intensities through blood, R = ln( $I^{680}$)/ln( $I^{850}$) is the ratio, and $a_{1-9}$ are the fitted coefficients from the regression based on our in-vitro data collection. The model was obtained by considering a function library consisting of polynomials of the input data and using elastic net regression with a 5-fold cross-validation to determine the optimal coefficients [31]. Often calibration-based models simply assume a polynomial model relating intensities to Hgb concentration without consideration of optimality [23], [24]. Using a generic sparse regression approach [32] we can maintain a high degree of fidelity with a minimum number of inputs. We selected the elastic net regression as opposed to ordinary least squares as it imposes sparsity (selecting only the most important predictors) and performs well when there are correlations between predictors [33]. Using the estimates of Hgb from eq. 2, we calculated the hemoglobin oxygen saturation based on eq. 3-5,

\begin{align*} {\mathrm {SO}}_{2}& = \alpha \cdot R + \beta \tag {3}\\ \alpha & = \mathrm {m}_{\alpha }\mathrm {\cdot Hgb + }\mathrm {b}_{\alpha } \tag {4}\\ \beta & = \mathrm {m}_{\beta }\mathrm {\cdot Hgb + }\mathrm {b}_{\beta } \tag {5}\end{align*}

where m ${}_{\alpha }$, m ${}_{\beta }$, b ${}_{\alpha }$, and b ${}_{\beta }$ are coefficients to be fitted for.

Equation 3 reflects the fact that the ratio R is highly linear with oxygen saturation [24]. However, the slope ( $\alpha $) as well as intercept ( $\beta $) are influenced by hemoglobin concentration (eq. 4-5).

We examined hemoglobin concentrations ranging from 6- $14\pm 0.5$ g/dL and oxygen saturations ranging from 50-100% to extract the coefficients for calibration and model validation. We assessed the model by computing the R^2^ and the adjusted R^2^, and generated Bland-Altman plots to assess the agreement between our optical sensors and the blood gas analyzer measurements [34]. Additionally, we computed the accuracy root-mean square error (ARMS) as a metric of device performance (table 2). A total of 86 measurements were collected from seven days of experiments and were split into two groups: calibration and validation. The calibration group was used to fit for the coefficients in our model and serve as reference for reproducibility within the same sensor and model stability. The validation group was used to test the model on unseen data and for computing the combined accuracy for Hgb and SO_2_ of the sensors.

**TABLE 1:** Calibration coefficients for Hgb

${}^{\mathrm {a}}1$	${}^{\mathrm {a}}2$	${}^{\mathrm {a}}3$	${}^{\mathrm {a}}4$	${}^{\mathrm {a}}5$	${}^{\mathrm {a}}6$	${}^{\mathrm {a}}7$	${}^{\mathrm {a}}8$	${}^{\mathrm {a}}9$
1.08	1.04	-28.08	0.70	-28.97	65.29	0.74	-13.40	118.95

**TABLE 2:** Sensor Accuracy Statistics

	Sensor 1			Sensor 2		
Parameter	${}_{\mathrm {R}}2$	Ad. R^2^	ARMS	${}_{\mathrm {R}}2$	Ad. R^2^	ARMS
Hgb (calibration)	0.	0. 3	1.28 g dL	0.84	0.80	1.12 g dL
Hgb (validation)	0. 8	0. 2	1.30 g dL	0.	0. 3	1.2 g dL
SO_2_ (calibration)	0.85	A	5.45 %	0. 3	A	3. 0 %
SO_2_ (validation)	0.82	A	5. 4 %	0. 2	A	3. 8 %

## III. Results

We first evaluate the intensity measured for the two sensors in the calibration data set. We found that due to manufacturing differences in the sensors the output intensity for the same wavelength differs across the two sensors. To account for this difference, we computed the average normalized intensity of the sensors per wavelength to be used as the inputs to the empirical calibration. We can then apply appropriate scaling factors to each sensor to enable the use of a single (sensor independent) calibration curve. Specifically, using equation (2) and fitting the scaled intensity data to the 9 unknown variables resulted in calibration coefficients listed in table 1.

**FIGURE 5.**
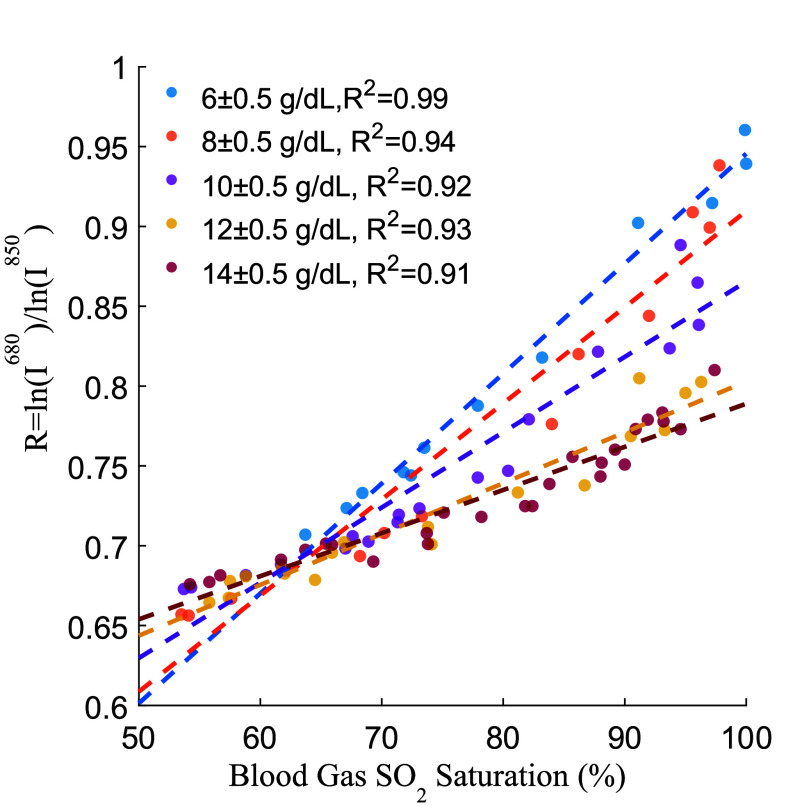
The ratio R as a function of oxygen saturation at different hemoglobin concentrations. The dashed lines are linear fits to the data for each concentration. The ratio is strongly linearly correlated with saturation; however, the slope and intercept are influenced by concentration.

**FIGURE 6.**
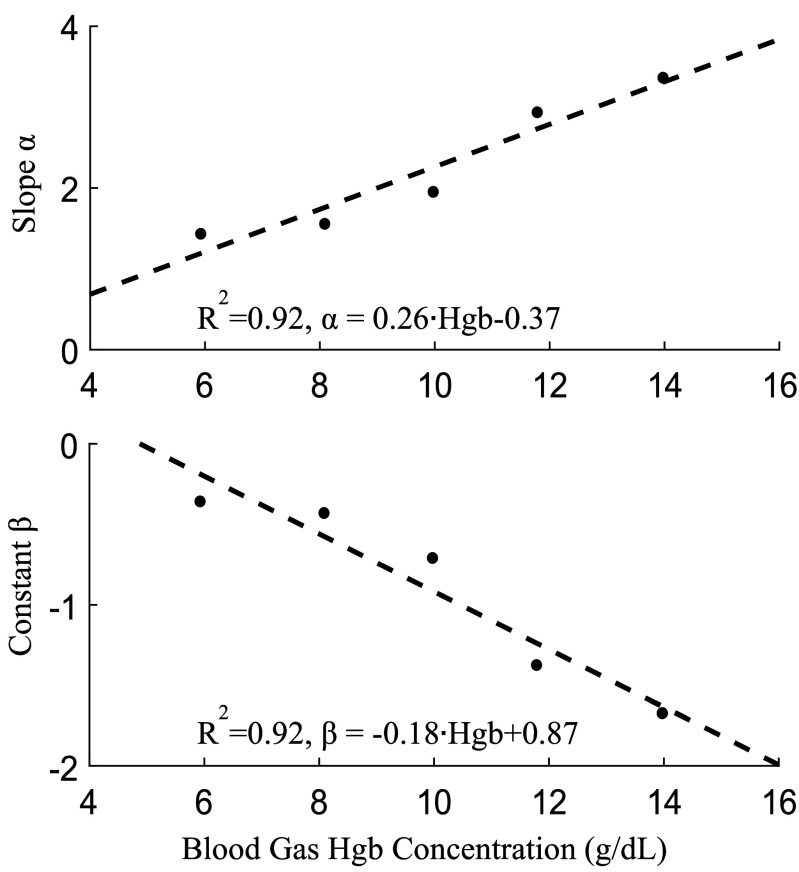
The slope ( $\alpha $) and intercept ( $\beta $) are influenced by hemoglobin concentration. Linear fits are applied to the data to correct for this effect with high R^2^ for both parameters.

**FIGURE 7.**
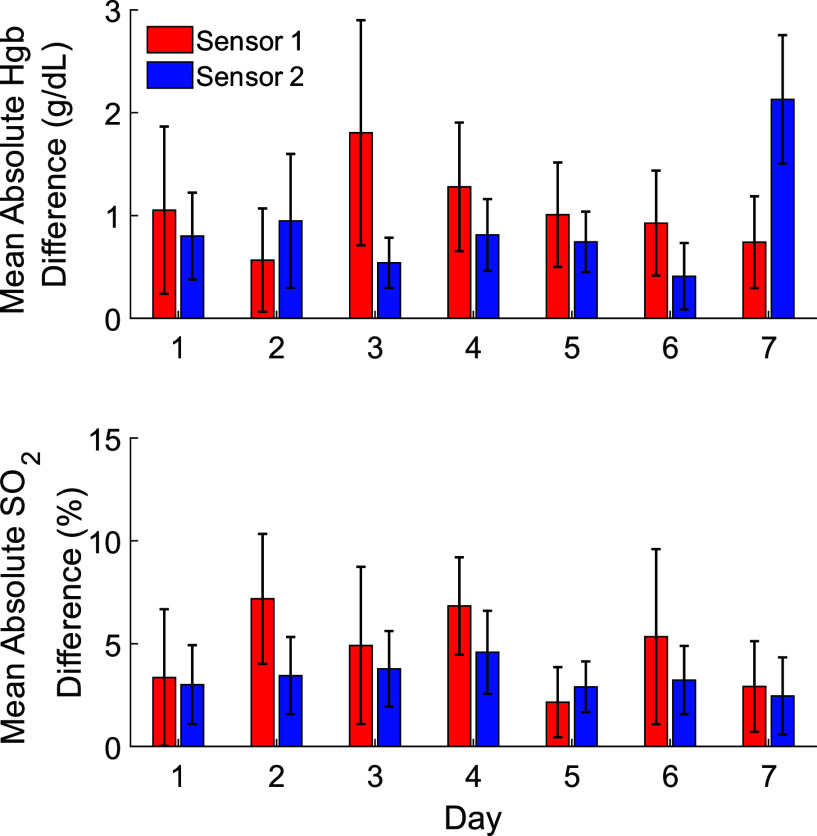
The average absolute differences between the sensor and blood gas measurements from the validation data across seven days.

**FIGURE 8.**
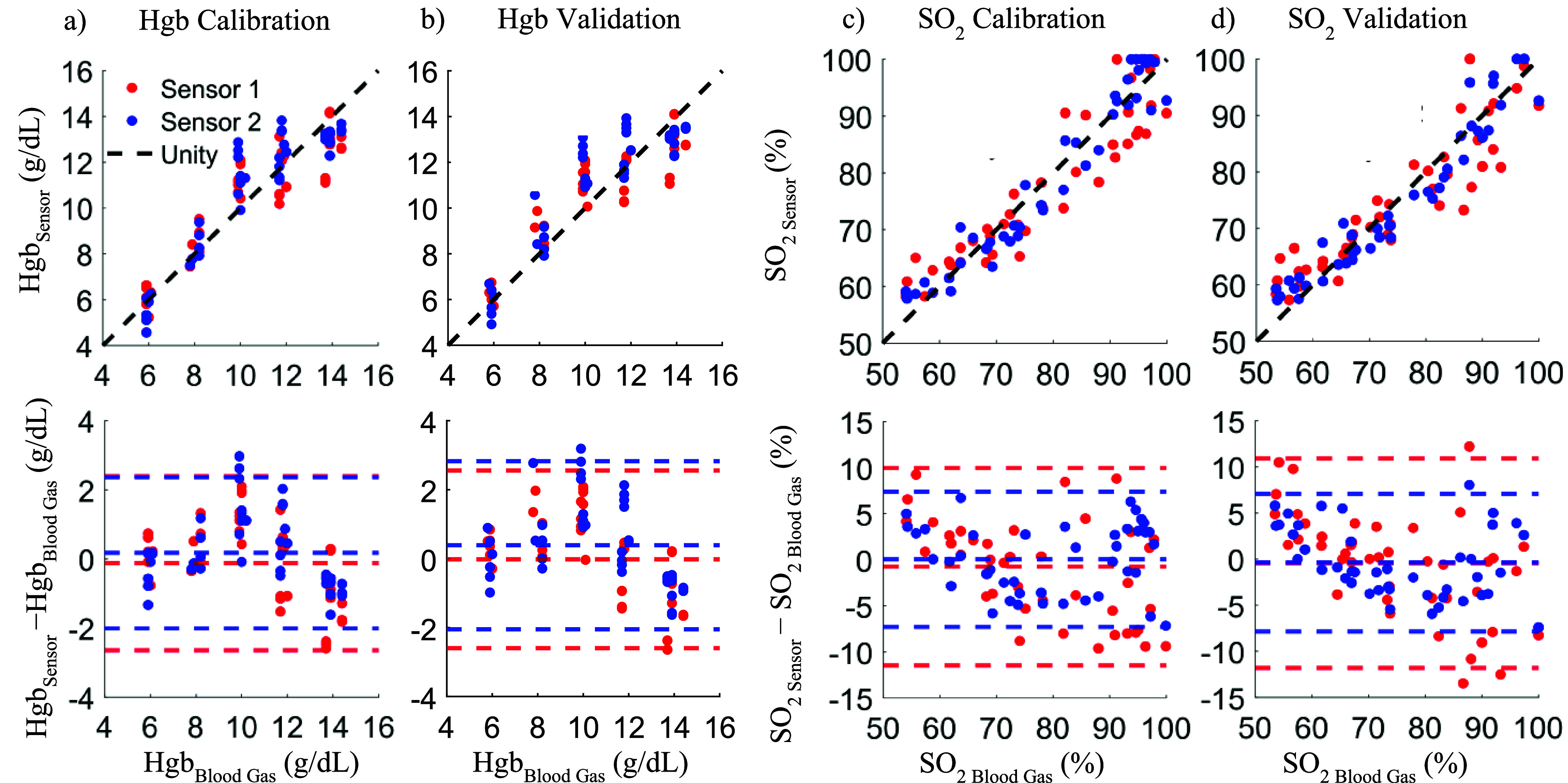
The predicted hemoglobin concentration and oxygen saturation from the two sensors are plotted against blood gas measurements (upper plots) and Bland-Altman plots are generated to assess their accuracy(bottom plots). In the Hgb plots (8a-b), the two sensors demonstrate similar performance with limits of agreements being approximately ±2 g/dL across calibration and validation data sets. In the SO_2_ plots (8c-d), sensor 2’s limits of agreement ( $\sim \pm 5$%) are narrower than sensor 1’s( $\sim \pm 10$%) but these differences are not statistically significant.

The same scaled intensity data as well as calculated Hgb is used with equations 3-5 to extract the unknown slope ( $\alpha $) and intercept ( $\beta $). The intensity ratio is plotted against oxygen saturation at different binned Hgb values (±0.5 g/dL) and linear fits were applied to the data (Figure 5). We found a strong linear correlation between intensity ratio and oxygen saturation at each concentration (R ${}^{2}> 0.9$). However, the slope and intercept of this relationship change linearly (R ${}^{2}= \,\, 0.92$) with increasing hemoglobin concentrations as seen in figure 6. This result is consistent with previous literature [24]. Using the obtained linear equations for $\alpha $ and $\beta $ (Fig. 6) combined with the estimated Hgb values allowed us to calculate oxygen saturation. The comparison of calculated versus ground truth hemoglobin concentration and oxygen saturation values and their deviations are shown in figures 7 and 8. Figure 7 shows the MAD of the blood gas measurements and the readings from our sensors across seven days. Table 3 shows the corresponding statistical results from the linear mixed model fit.

**TABLE 3:** Linear Mixed Results

Parameter (Respons e)	Coefficient	Standard Error	^p^	95% C
ntercept (Hgb)	0.83	0.124	0.001	0.5 3, 1.08
Day (Hgb)	0.03 8	0.0265	0.135	-0.0125, 0.0 21
ntercept (SO_2_)	4.53	0.4 8	0.001	3.5, 5.4
Day (SO2)	-0.158	0.102	0.123	-0.35, 0.0431

Results are shown for data used for calibration (8a and 8c) as well as validation (8b and 8d). The upper row of figure 8 shows scatter plots of calculated versus ground truth hemoglobin concentration and oxygen saturation, while the bottom row shows the Bland-Altman plot for the same data sets. Color in figure 8 shows two different sensors. The R^2^, adjusted R^2^, and ARMS for each sensor and group per parameter (Hgb and SO_2_) are computed and reported in table 2.

## IV. Discussion and Conclusion

We presented an approach for developing and calibrating optical sensors for real-time monitoring of oxygen saturation and hemoglobin concentration in whole blood. Results showed an average ARMS (accuracy) of 1.30 g/dL for concentration and 4.76 % for saturation across the two devices. The consistency of the R^2^ and adjusted R^2^ across the calibration and validation groups is indicative of model stability and a lack of overfitting. The calibration and validation group results for hemoglobin concentration extraction are similar for sensor 1 as indicated by their R^2^ (0.79 and 0.78 respectively), adjusted R^2^ (0.73 and 0.72 respectively) and ARMS (1.28 g/dL and 1.30 g/dL respectively). The results for sensor 2 worsen from calibration (R ${}^{2}=0.84$, adjusted R ${}^{2}=0.80$, ARMS= 1.12 g/dL) to the validation group (R ${}^{2}=0.79$, adjusted R ${}^{2}=0.73$, ARMS= 1.29 g/dL). However, this change is not statistically significant. The similarity between sensors is further demonstrated by Bland-Altman plots when comparing the mean biases and limits of agreement. For determining oxygen saturation, both sensors show similar results across the calibration and validation groups. Sensor 2 demonstrates better overall calibration (R ${}^{2}=0.93$, ARMS= 3.70%) and validation performance (R ${}^{2}=0.92$, ARMS= 3.78%) for extraction of oxygen saturation compared to sensor 1’s calibration (R ${}^{2}=0.85$, ARMS= 5.45%) and validation results (R ${}^{2}=0.82$, ARMS= 5.78%). Additionally, the limits of agreement are narrower for sensor 2 compared to sensor 1. However, these differences between the sensors are not statistically significant, demonstrating reproducibility across devices.

To demonstrate comparability to commercial monitors, we compare the limits of agreements (LOA) of our devices from the validation data to those obtained from clinical cases using the Terumo and Spectrum monitors. For the Spectrum M4, the LOA for Hgb are −3.204 and 3.256 g/dL, while for the CDI 500 the LOA are −1.803 and 2.153 g/dL [5]. The Hgb LOA for sensor 1 (-2.597, 2.557 g/dL) and sensor 2 (-2.049, 2.826 g/dL) fall between the LOAs of the commercial systems providing an indication of similar performance. This indication is further supported by comparing LOAs for SO_2_.

For the Spectrum M4 and Terumo, the LOAs are −12.741 and 10.327 %, and −12.503 and 10.362 % respectively. In comparison, sensor 1’s LOA were -11.809 and 10.903 %, while sensor 2’s LOA were -7.837 and 7.080 %. Follow-up studies comparing clinical devices to our sensors are needed to further support these results.

Additionally, the MAD results from figure IV for oxygen saturation and hemoglobin concentration show no clear trend across days across the two sensors. There are two observable outliers for hemoglobin concentration on day 3 and day 7 with sensor 1 and sensor 2 respectively. However, errors still fall within the level of deviations observed in clinical cases with commercial monitors. Furthermore, we found no statistically significant differences across the days as shown by table 3.

While we have seen good agreement between sensors, each sensor accuracy is dependent on calibration accuracy. Given that SO_2_ and Hgb are coupled, we do expect a certain amount of cross talk. Additionally, the Bland-Altman plots, particularly for Hgb, demonstrate deviations from blood gas values that are non-linear making the error in sensor measurements dependent on the true Hgb concentration. One possible way to mitigate these effects is through the inclusion of an additional wavelength at an isosbestic point (for instance near 800 nm). Use of additional wavelengths can reduce overall error in the sensor and remove the observed non-linearity [11]. Furthermore, since intensity changes at the isosbestic wavelength are only affected by Hgb concentration, cross talk between SO_2_ and Hgb could be minimized.

Our work here was performed solely through controlled in-vitro studies with bovine blood. As such, we lack data on the performance of our sensors in clinical cases. Although the absorption properties of hemoglobin are the same between bovine and human blood [35], the effect of other hematological factors (flow changes, plasma composition, red blood cell size and shape, etc.) on sensor readings was not assessed. Changes in blood temperature could influence sensor readings due to variations in blood optical properties [36], [37]. However, this is not a concern as patient blood temperature in ECMO is typically well-controlled using heat exchangers built into the gas exchanger. Other factors like tubing material and size would impact the sensor readings as our sensors were calibrated specifically for transparent Tygon tubing at 3/8” inner diameter. Sensors would need to be recalibrated to accommodate other conditions and settings. Additionally, we have not examined the temporal stability of the calibration over an extended period. Thus, recalibration of the sensors may be necessary. Future work will focus on assessing the impact of these factors on the sensors and their translatability through long-term (30-day) in-vivo studies on an ECMO circuit.

Our sensors have demonstrated potential for monitoring patient hemodynamics and aiding in clinical decisions of blood transfusion [38], [39], [40], [41]. They could also be used to assess ECMO oxygenator functionality in real-time through pre- and post-oxygenator blood saturation and oxygen delivery rate [40], [41], [42], [43], [44]. While clinically available hemoglobin concentration and oxygen saturation monitors comparable to our sensors do exist (e.g. Terumo CDI 500 and Spectrum M4 monitor), our devices are of a small form factor reducing the bulk that commercial monitors contribute to extracorporeal systems such as ECMO [3]. Another advantage our sensors have over current devices is that they read through standard Tygon tubing forgoing the need for a specialized connector. This aspect is valuable since these custom connectors often disrupt flow in extracorporeal systems and are procoagulant. By developing a clamp-on device instead of an inline one, our device avoids these risks altogether. The portability aspect of the developed sensors allows for effortless translation into the clinic. Additionally, setup of the devices can be performed within a few minutes and have been developed to be compatible with medical grade tubing. These sensors possess the potential to allow for translation of other miniaturized systems (e.g. PAS), while remaining compatible with existing systems. Future in-vivo studies and clinical studies will need to be performed to assess their reliability and potential for replacement of current monitoring devices.

We conclude that based on the presented work our sensors and calibration possess advantages over existing devices, however, further studies assessing their performance in clinical settings are needed for demonstrated reliability and translation.
